# A probable role of copper in the comorbidity in Wilson’s and Creutzfeldt-Jakob’s Diseases: a case report

**DOI:** 10.1186/s12985-020-01309-x

**Published:** 2020-03-13

**Authors:** Effrosyni Koutsouraki, Dimitrios Michmizos, Olga Patsi, John Tzartos, Martha Spilioti, Marianthi Arnaoutoglou, Magda Tsolaki

**Affiliations:** 1 Aristotle University, 1st Neurology clinic, AHEPA Hospital, Thessaloniki, Greece; 2 University of Thessaly, Volos, Greece; 3 Tzartos Neurodiagnostics, Athens, Greece

**Keywords:** Wilson’s disease, Copper, Creutzfeldt-Jakob disease, Prion protein, Spongiform encephalopathy

## Abstract

**Background:**

To the best of our knowledgedd, there is currently no case in the literature reporting the comorbidity of Wilson’s and Creutzfeldt-Jakob disease (CJD), linked through copper.

**Case presentation:**

A 44-year-old male with a history of inherited Wilson’s disease (hepatolenticular degeneration), which manifested as mild liver injury and psychiatric symptoms, was admitted to our department due to speech and cognitive disturbances. Upon his admission, he had motor aphasia as well as psychomotor retardation with an otherwise normal neurological examination. Laboratory tests, including liver enzymes, copper and serum ammonia were all within normal range. The brain MRI showed increased T2 signal in the caudate nuclei, attributed to copper deposition in the context of Wilson’s disease. In the electroencephalogram, periodic sharp discharges were eminent, initially unilateral and then generalized. The positive 14–3-3 protein in the cerebrospinal fluid (CSF) and the new brain MRI, that demonstrated elevated DWI signal not only in the basal ganglia but also in parts of the cerebral cortex (cortical ribbon sign), all supportive of a possible CJD diagnosis. The detection of PrP^Sc^ in the patient’s CSF, using the RT-QuIC method, which has a 99.4–100% specificity for CJD, made the diagnosis of CJD highly probable.

**Conclusion:**

This is the first report of Wilson’s and Creutzfeldt-Jakob diseases co-morbidity in the literature, which could evoke a possible role of copper in the pathogenesis of CJD.

## Background

Although the precise function of PrP^C^ in healthy tissues is not yet known, recent research has demonstrated that it binds copper (Cu) in an unusual and highly conserved region of the protein termed the octarepeat domain [1, 2]. A possible synergistic role of the prion protein in the pathologic metabolism of copper occurring in the context of Wilson’s disease has also been suggested [3, 4]. The transmissible spongiform encephalopathies (TSEs) arise from the conversion of the membrane-bound prion protein from PrP^c^ to PrP^Sc^. Examples of the TSEs include mad cow disease, chronic wasting disease in deer and elk, scrapie in goats and sheep, and kuru and Creutzfeldt-Jakob disease (CJD) in humans. Sporadic and inherited prion diseases fall into the same class as Alzheimer’s and Parkinson’s disease, insofar that they are all associated with the accumulation of endogenous protein aggregates [5–9].

We present a case of co-morbidity of hepatolenticular degeneration (Wilson’s disease) and CJD that suggests a possible association of copper metabolism with the pathogenesis of CJD disease.

## Case presentation

A 44-year-old male with a known history of inherited Wilson’s disease (both his brother and father suffered from the same disease), who had mild liver problems for 12 years and bipolar psychosis in the months prior to presenting himself, was admitted to the emergency services of our University neurological department with a month-long progressive episode of aphasia.

The diagnosis of Wilson’s disease was based on the patients’ metabolic profile, the increased urine copper excretion combined with decreased ceruloplasmin plasma levels**.** Psychiatric symptoms were present from the initial states of the disease. Almost all family members showed agitation or bipolar disorder. Liver pathology relevant to the disease was present in both siblings whereas the father’s liver biopsy was normal. No focal neurological deficit was present at the time diagnosis was established in all three men. PCR testing for the most prevalent ATP78 variants in the Greek population was negative; however, Sanger sequencing analysis was not performed.

**Fig. 1:**
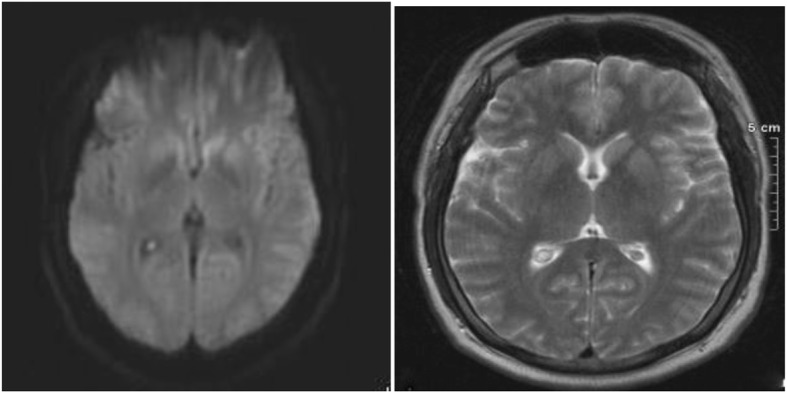
Brain MRI revealed increased DWI and T2 signal in the caudate nuclei (left>right)

**Fig. 2:**
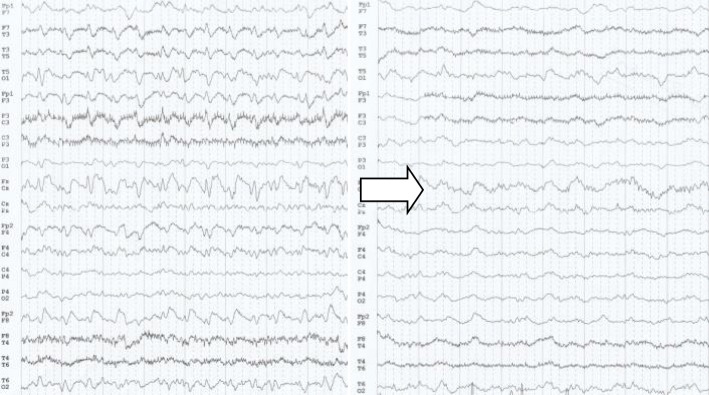
EEG: Lateralized periodic discharges -LPDs stopped by intravenous benzodiazepines

Upon his admission to our clinic, he had motor aphasia and psychomotor retardation without any other neurological signs and with a Glasgow Coma Scale score of 13/15 (5-4-4). Laboratory tests, including liver enzymes, copper and serum ammonia were all normal, whereas the brain MRI showed an increased T2 signal in the caudate nuclei attributed to copper deposition in the context of Wilson’s disease (Fig. 1). The EEG revealed slowing and lateralized periodic discharges, slow (< 2.5 Hz) spikes and waves over the left hemisphere (less often involving the right one) with spatiotemporal evolution. These EEG findings raised the suspicion of non-convulsive status epilepticus, thus they were suppressed by iv benzodiazepines but without clinical improvement (Fig. 2) and antiepileptic treatment with iv levetiracetam was initiated.

CSF examination showed normal cell count, glucose and protein levels and there was no intrathecal viral antibody production. The CSF culture was negative while negative was also the search for antibodies to an autoimmune encephalitis antigen panel.

The patient’s clinical condition deteriorated very rapidly. As his aphasia evolved to mutismus, he developed myoclonic jerks and there was gradual worsening of his level of consciousness. Finally, within a few days, the patient demonstrated myoclonic status epilepticus which remained unresponsive to benzodiazepines or other antiepileptic treatment. An empirical treatment with high-dose iv methylprednisolone for 7 days was also administered with no benefit. The rapid cognitive deterioration and the myoclonic status raised the hypothesis of CJD.

**Fig. 3:**
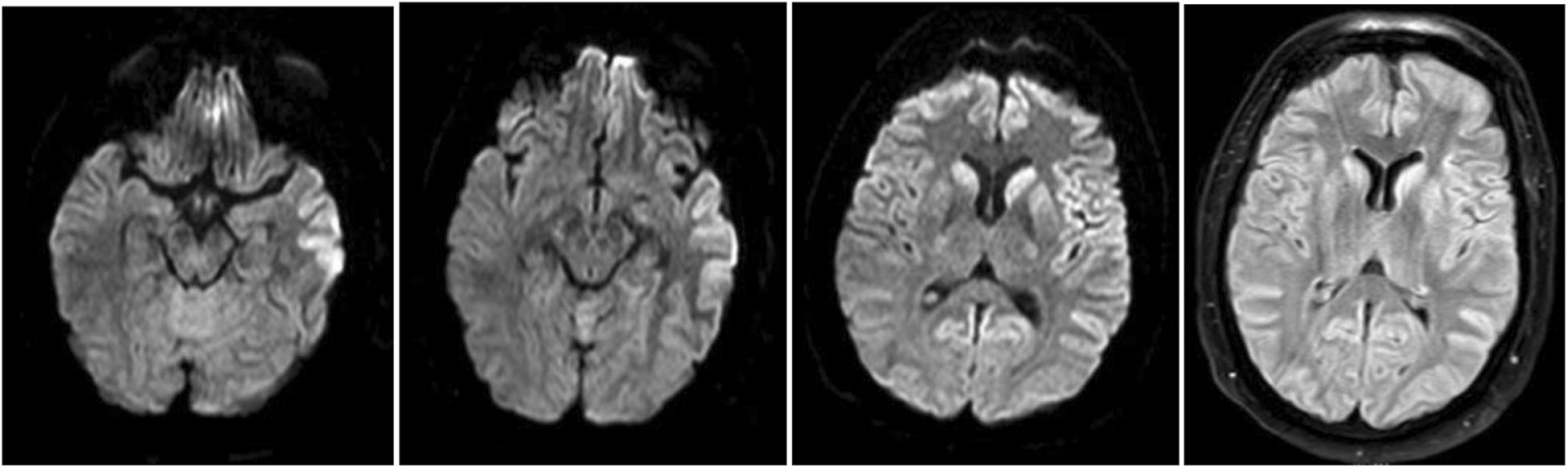
Second brain MRI showing increased DWI and T2 (FLAIR) signal in basal ganglia and cerebral cortex (cortical ribbon sign)

**Fig. 4:**
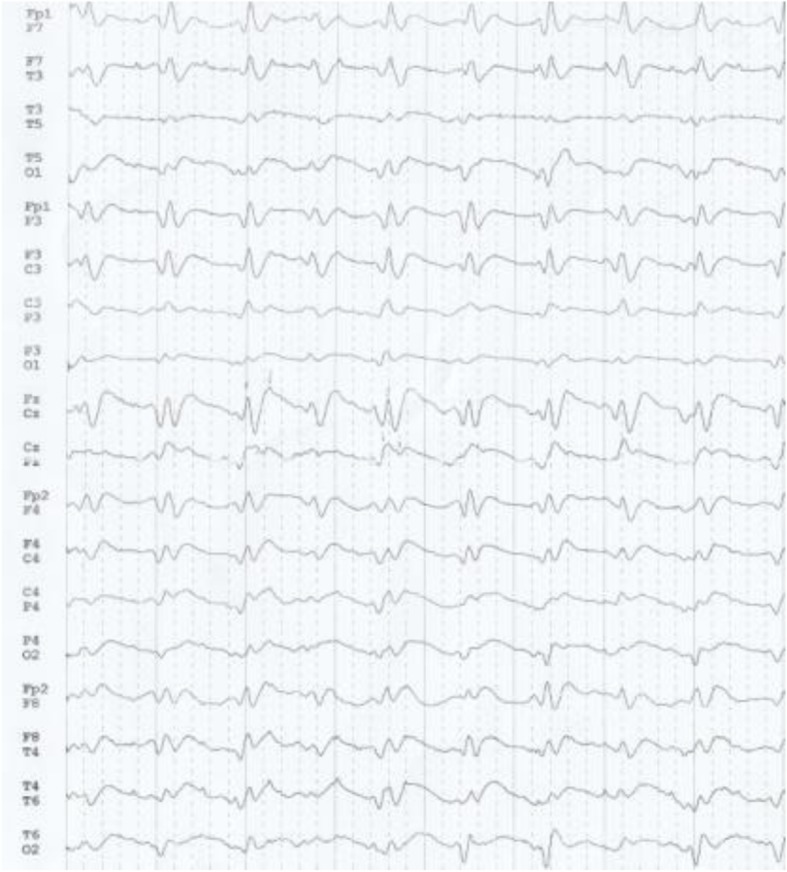
New EEG showed periodic sharp wave complexes (PSWC) suggesting CJD

A second brain MRI, performed 15 days later, showed increased DWI and T2(FLAIR) signal not only in the basal ganglia but also in the cerebral cortex (cortical ribbon sign), also suggesting CJD [10, 11] (Fig. 3). A second EEG showed periodic sharp wave complexes (PSWC) with predominance over the left frontocentral regions demonstrating the triphasic morphology, typical of CJD (Fig. 4).

Finally, a second CSF examination revealed positive values for all CSF biomarkers tested: protein 14–3-3 (CircuLex ELISA, MBL, Japan), Tau and phosphoTau (Fujirebio/Innotest ELISA) and PrP^Sc^ detection by RT-QuIC [12, 13] (Table 1).

**Table 1:** CSF biomarkers

Test	Result
14–3-3	Positive, 166,000 AU/ml
Tau	1920 pg/ml (> 1150 increases CJD probability)
P-tau	18 pg/ml
Tau/p-Tau ratio	106 (> 17 increases CJD probability)
PrP^Sc^(RT-QulC) [12, 13]	Positive

The clinical picture, the highly positive 14–3-3 protein, Tau and Tau/pTau levels in the CSF and the new brain MRI that demonstrated elevated DWI signal not only in the basal ganglia but also in the cerebral cortex (cortical ribbon sign) all made the diagnosis of CJD possible. The detection of PrP^Sc^ in the CSF using the RT-QuIC method, which has been reported to have a 99.4–100% specificity for CJD [12, 13], made the diagnosis of CJD highly probable [14].

The patient kept deteriorating and passed away 2 months after his initial presentation. At the request of the family no autopsy was performed.

## Discussion and conclusions

The present case may well be the first report of Wilson’s and Creutzfeldt-Jakob diseases co-morbidity, suggesting a possible association of copper metabolism with the pathogenesis of CJD. Several recent studies investigate the relationship of copper and prion protein and its role in the pathogenesis of the CJD which remains unclear [7–9]. This case sparks a new clinical interest in pursuing further research in this direction.

## Data Availability

The datasets used during the current study are available from the corresponding author on reasonable request.

## Abbreviations

CJD: Creutzfeldt-Jakob disease
CSF: Cerebrospinal fluid
Cu: Copper
DWI: Diffusion-Weighted Image
EEG: Electroencephalogram
ELISA: Enzyme linked immunosorbent assays
FLAIR: Fluid-Attenuated Inversion Recovery
iv: Intravenous
MRI: Magnetic resonance imaging
PCR: Polymerase chain reaction
PrP^Sc^: Scrapie isoform of the prion protein
PrP^c^: Cellular prion protein: Real-time quaking-induced conversion
PSWC: Periodic sharp wave complexes
RT-QuIC: Real-time quaking-induced conversion
TSEs: Transmissible spongiform encephalopathies

## References

[1] Qin K, Coomaraswamy J, Mastrangelo P (2003). The PrP-like protein Doppel binds copper. J Biol Chem.

[2] Aronoff-Spencer E, Burns CS, Avdievich NI (2000). Identification of the Cu2+ binding sites in the N-terminal domain of the prion protein by EPR and CD spectroscopy. Biochemistry.

[3] Merle U, Stremmel W, Geßner R (2006). Influence of Homozygosity for methionine at codon 129 of the human prion gene on the onset of neurological and hepatic symptoms in Wilson disease. Arch Neurol.

[4] Forbes N, Goodwin S, Woodward K (2014). Evidence for synergistic effects of PRNP and ATP7B mutations in severe neuropsychiatric deterioration. BMC Med Genet.

[5] Millhauser GL (2007). Copper and the Prion Protein: Methods, Structures, Function, and Disease. Ann Rev Phys Chem.

[6] Wulf M-A, Senatore A, Aguzzi A (2017). The biological function of the cellular prion protein: an update. BMC Biology.

[7] Yen C-F, Harischandra DS, Kanthasamy A, Sivasankar S (2016). Copper-induced structural conversion templates prion protein oligomerization and neurotoxicity. Sci Adv.

[8] Stevens DJ, Walter ED, Rodríguez A, Draper D, Davies P, Brown DR, Millhauser GL (2009). Early onset prion disease from Octarepeat expansion correlates with copper binding properties. PLoS Pathog.

[9] Evans EG, Millhauser GL. Copper-and Zinc-Promoted Interdomain Structure in the Prion Protein: A Mechanism for Autoinhibition of the Neurotoxic N-Terminus. In: Progress in molecular biology and translational science, vol. 150.: Academic Press; 2017. p. 35–56. 10.1016/bs.pmbts.2017.06.005 28838668

[10] Brown HG and Lee JM. Creutzfeldt-Jakob disease. Aminoff MJ, ed. UpToDate. Waltham, MA: UpToDate Inc http://www.uptodate.com Accessed 2 May 2018.

[11] Abdulmassih R, Min Z (2016). An ominous radiographic feature: cortical ribbon sign. Intern Emerg Med.

[12] McGuire LI, Peden AH, Orrú CD, Wilham JM, Appleford NE, Mallinson G, Green AJ (2012). RT-QuIC analysis of cerebrospinal fluid in sporadic Creutzfeldt-Jakob disease. Ann Neurol.

[13] Bongianni M, Orrù C, Groveman BR, Sacchetto L, Fiorini M, Tonoli G (2017). Diagnosis of human prion disease using real-time quaking-induced conversion testing of olfactory mucosa and cerebrospinal fluid samples. JAMA Neurology.

[14] Hermann P, Laux M, Glatzel M (2018). Validation and utilization of amended diagnostic criteria in Creutzfeldt-Jakob disease surveillance. Neurology.

